# Plate Augmentation in Aseptic Femoral Shaft Nonunion after Intramedullary Nailing: A Literature Review

**DOI:** 10.3390/bioengineering9100560

**Published:** 2022-10-16

**Authors:** Carlo Perisano, Luigi Cianni, Chiara Polichetti, Adriano Cannella, Massimiliano Mosca, Silvio Caravelli, Giulio Maccauro, Tommaso Greco

**Affiliations:** 1 Department of Ageing, Neurosciences, Head-Neck and Orthopedics Sciences, Orthopedics and Trauma Surgery, Fondazione Policlinico Universitario Agostino Gemelli IRCCS, 00168 Rome, Italy; 2 Orthopedics and Trauma Surgery, Università Cattolica del Sacro Cuore, 00168 Rome, Italy; 3 IRCCS Istituto Ortopedico Rizzoli—U.O.C. II Clinic of Orthopaedics and Traumatology, 40136 Bologna, Italy

**Keywords:** femoral shaft fracture, nonunion, plate augmentation, augmentative plate, intramedullary nailing, plate fixation

## Abstract

Background: Femoral shaft fractures (FSFs) are a frequent injury in traumatology for which intramedullary nailing (IMN) is considered the gold standard treatment. Nonunion (NU) is one of the most frequent complications in FSF treated with IMN, with a percentage from 1.1% to 14%. Plate augmentation (PA), the addition of a compression plate and screws, with or without bone graft has been described as an effective option for the treatment of NU, improving the biomechanical conditions at the fracture site. The aim of this review was to analyze the literature relating to the use of PA in NU after IMN in FSFs to assess the efficacy of the technique. Methods: An electronic search on PubMed, Google Scholar, and Web of Science was conducted to search for all studies concerning PA of femoral shaft NUs after IMN. Results: Twenty-four studies were included in the review comprising a total of 502 patients with a mean age of 39.5 years. Of these, 200 hundred patients had atrophic pseudoarthrosis and 123 had hypertrophic pseudoarthrosis, while in 179, the type of pseudoarthrosis was not reported. The most frequently used plate for PA was the dynamic compression plate (DCP); in 87.1% of the cases, the authors added a bone graft to the plate fixation. In 98.0% of the patients, a complete bone union was achieved in a mean time of 5.8 ± 2.12 months. Conclusion: The patients treated with PA included in this review showed a good rate of consolidation in the femoral shaft NUs, with good functional recovery and a low incidence of complications.

## 1. Introduction

Femoral shaft fractures (FSFs) are a frequent injury in traumatology, often caused by either high-energy trauma such as road traffic accidents or low-energy trauma such as fractures in the elderly due to osteoporosis [1].

Interlocked intramedullary nailing (IMN) is the gold standard treatment for FSFs, with good union results and a success rate of approximately 95% [2]. 

Nonunion (NU) is defined as incomplete healing within 9 months of injury or no evidence of healing on successive radiographs over 3 months; NUs are differentiated into hypertrophic NUs (also known as elephant foot NUs, characterized by exuberant callus formation due to inadequate biomechanical stability) and atrophic NUs (attributable to inadequate vascular supply to the fracture site or nonunion), or into septic and aseptic NUs (depending on the presence or absence of infection) [3,4].

Although an aseptic NU of the femoral shaft after IMN is rather rare compared to other bone localizations [5,6,7], recent studies suggest that it may occur in the range of 1.1% to 14% [8,9]. On average, 200 cases of long bone nonunion occur per million population, estimating a total of 150,000 cases in Europe each year [10].

The causes of fixation failure and the occurrence of NU may depend on both mechanical factors (such as insufficient stability due to small nail size, rotational instability, lack of locking, or malalignment in the upper or lower third fractures, especially with comminution) and biological factors (such as the severity of the bone injury and soft tissue damage, open fractures, extended comminution, wide displacement of fragments, interposition of fragments, smoking, diabetes, neuropathies, alcoholism, corticosteroids, malnutrition, or previous radiotherapy) [11].

However, the major risk factors seem to be instability at the fracture site and shear stress [12]. NU after IMN in an FSF is infrequent but challenging to treat, with a high impact on the patient’s quality of life, often requiring long treatments with multiple surgeries [2]. Several types of treatment are reported in the literature: nail removal followed by internal fixation with a plate and screws [13], reamed and exchange nailing [14], retaining the nail and plate augmentation (PA) [2], stable fixation with or without bone grafting, dynamization of the nail [15], and Ilizarov external fixation [16]. 

PA, i.e., the addition of a compression plate and screws, with or without bone graft has been described as an effective option for the treatment of NU, improving the biomechanical conditions at the fracture site without adding significant biological damage—rather, it gives the surgeon the opportunity to clean the fracture site of fibrous callus and freshen the fracture ends as a stimulus for healing; there is also the opportunity for direct bone grafting at the surgeon’s discretion [17,18].

The aim of this review was to analyze the existing literature to assess the efficacy and safety of PA treatment in NU after IMN in FSF.

## 2. Materials and Methods

A literature search was performed in agreement with the PRISMA (Preferred Reporting Items for Systematic Reviews and Meta-Analyses) [19] guidelines from June 2022 (PRISMA checklist in Supplementary Materials). The protocol was registered and allocated in the PROSPERO database (CRD42022363873), hosted by the National Institute for Health Research, University of York, Center for Reviews and Dissemination.

### 2.1. Search Strategy

A comprehensive literature search was conducted on MEDLINE through PubMed, Google Scholar, and Web of Science (WOS), using the search terms “femoral shaft nonunion”, “long bone nonunion”, “plate augmentation”, and “augmentative plate”, to identify relevant studies and publications concerning PA in FSF-NUs treated with IMN. 

To minimize the number of missed studies, no time restriction and no filters were applied to the search strategies. In addition, a manual search for references from review articles was performed to supplement the electronic database search.

### 2.2. Inclusion and Exclusion Criteria

Inclusion criteria were studies on PA in aseptic NUs of FSF previously treated with IMN, written in English, with full text available.

Exclusion criteria were studies on septic or infected NUs, NUs in femoral segment different from shaft, with unspecified surgical treatment, on animal models, or on NUs of long bones without reporting specific data on the femur. Case reports, literature reviews, editorial pieces, and studies not in the English language were also excluded.

From titles and abstracts, two authors (L.C., M.S.) independently selected studies for inclusion. In cases of disagreement of paper inclusion/exclusion at any stage of the selection process, a consensus was reached through discussion or, where necessary, by arbitration from the senior author. 

Titles of journals, names of authors, and supporting institutions were not masked at any stage. No attempt was made to contact authors to obtain individual patient data. 

### 2.3. Data Extraction

The following characteristics of the study and patients were collected: authors, year of publication, type of study, number of patients, sex, age, NU localization (proximal third, middle third, or distal third of femoral shaft), type of NU, use of bone graft (BG) or not, union rate, time to union, type of plate used, exchange of the nail or not, complications after surgery (broken screw, venous thrombo-embolic disease, wound infection, hardware infection, shortening lower limb), functional scores, and follow-up duration. Bone union was evaluated in all studies according to radiographic criteria.

The femoral shaft was considered the portion of the femur between 5 cm distal to the lesser trochanter and 5 cm proximal to the adductor tubercle.

Where possible, we divided the NUs into atrophic and hypertrophic according to the classifications used by the various authors.

Due to the heterogeneity of the publications analyzed in terms of patient samples and study designs, some of these values were not reported or impossible to extrapolate and were considered missing and not applicable in the presentation of our results. 

Two authors (L.C., M.S.) independently extracted the available data from the full text of all eligible studies using a pilot form. Two other investigators (T.G., C.P.—Chiara Polichetti) checked the accuracy of the extracted information. 

Statistical analysis was performed using SPSS 18.0 for Windows (SPSS Inc., Chicago, IL, USA). Descriptive statistics were used to summarize the findings across all of the included studies; a meta-analysis was not possible due to the high heterogeneity of the included studies.

## 3. Results

**Figure 1:**
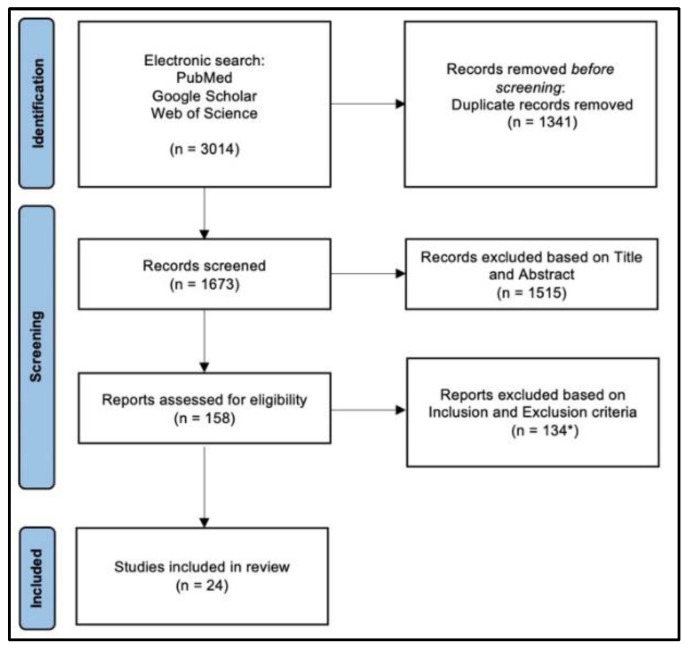
PRISMA flow-chart. (* 43 = studies on femoral NUs in segments other than the shaft, 39 = studies without surgical data, 37 = studies on septic NUs, 15 = studies on animal models).

After literature research, 3014 papers were selected for further evaluation. The removal of duplicated titles left 1673 studies. Of these, 1515 were omitted because the title or abstract did not fulfil the inclusion criteria. The remaining 158 studies were analyzed in detail. Finally, 24 studies fit our inclusion criteria and were included in the study [2,8,11,12,17,18,20,21,22,23,24,25,26,27,28,29,30,31,32,33,34,35,36,37] (Figure 1).

Out of 24 selected studies, 21 were retrospective [2,8,11,12,17,18,20,21,22,23,24,25,26,27,28,29,30,31,32,36,37] and 3 prospective [33,34,35] (Table 1).

### 3.1. Demographics

Overall, data from 502 patients were analyzed. In 38 patients, sex was not specified [22,36]; in the remaining 479, 347 were male (74.8%) and 117 female (25.2%) (Table 2).

The average age of the population was 39.5 ± 5.4 years old; only one study of about 5 patients did not report the average age [32].

In 12 studies [12,17,18,20,21,24,26,27,28,31,34,37], the NU localization was reported. For a total of 314 patients, 53.5% of NUs (168 cases) were in the middle third of the diaphysis, 31.5% (99 cases) in the distal third, and 15.0% (47 cases) in the proximal third. The type of NU was specified in 13 articles [2,8,12,17,18,20,24,25,26,28,30,31,37], with a total of 323 patients. The cases were atrophic NUs in 200 cases (61.9%) and hypertrophic NUs in 123 cases (38.1%).

For the acute treatment of initial FSFs, anterograde nailing was used in in 151 patients (85.8%) and retrograde in 25 patients (14.2%), while the type of nailing used was not reported for 326 patients.

### 3.2. Surgical Information about PA

For PA, all the authors used a direct lateral approach, regardless of the location in the femoral shaft. In 287 patients, surgery was performed using a dynamic compression plate (DCP), in 109 patients using a locking compression plate (LCP), in 65 patients using a limited contact–dynamic compression plate (LC–DCP), and in 2 patients using a proximal humeral anatomic locking plate (PHLP). In one study, both plates (DCP and LC–DCP) were used but without specifying the number of patients [28].

In three studies [29,31,33], representing 106 patients, a nail exchange was also performed before PA. In contrast, Jhunjhunwala and Dhawale [18] performed a nail exchange in only 9 cases in their cohort of 40 patients. In total, therefore, 115 patients underwent nail exchange followed by PA.

Bone graft from the iliac crest was used in a total of 437 patients (87.1%), and in one study [17], bone morphogenic proteins (BMPs) were used in addition to the bone graft.

Where possible, data were collected on the number of screws used, surgery time, and blood loss. The average number of screws used was 4.1 proximal (±1.07) and 4.2 distal (±1.2). The average surgical time was 83.4 min (±13.02). The average amount of blood loss was 257.2 mL (±72.12).

### 3.3. Outcomes

A total of 492 out of 502 patients (98.0%) achieved bone consolidation, in an average time of 5.8 months (±2.12) (Table 3).

In 18 studies, a bone union of 100% [2,11,20,21,22,23,24,24,25,27,29,30,31,32,33,34,35,37] was reported; in 4 studies, bone union was reported between 95% and 100% [17,18,28,36]; and in 2 studies, bone union was between 85% and 90% [8,12].

Weight bearing was granted after an average of 6.2 weeks (±8.27 weeks), and the mean follow-up time was 18.2 months (range 9.6–42.3).

For functional scores, a preserved ROM of the hip and knee was reported in 17 studies [2,8,11,18,20,21,22,23,24,25,26,27,29,31,33,34,35], while in one study [36], the decrement in the active knee range of motion was more than 20% compared with the opposite side in 47.4% of patients. No functional results were reported in 6 studies [12,17,28,30,32,37].

A total of 45 complications were reported. The most frequent complication was a shortening of the limb in 25 patients; in 13 of these, the shortening was approximately 1 cm, while in the remaining 12 patients, the shortening was greater than 1 cm and caused walking impairment. Other complications were wound infection in 10 patients, graft site donor (iliac crest) pain in 3 patients, a broken screw in 2 patients, deep venous thrombosis in 2 patients, deep infection in 2 patients, and infected hematoma in 1 patient. In the 2 patients with deep infection, it was not specified which type of initial NU was presented, but a DCP plate was used in both; in the first case, the infection was treated with the removal of the implants and EN with a vancomycin-coated nail, and union was achieved after 1 year; the second case, on the other hand, healed after the removal of the hardware and administration of intravenous antibiotics.

## 4. Discussion

The aim of this review was to analyze the efficacy and safety of using PA in femoral shaft NUs following FSF treated with IMN. Although IMN is the treatment of choice for FSF, with a good bone union rate, between 1.1% and 14% of IMN-treated FSFs can be complicated by NU (estimated at approximately 150,000 cases each year in Europe) [2]. 

The causes of failure may depend on mechanical and biological factors [25,38,39]. In the literature, the treatments proposed for femoral shaft NU treated with IMN range from exchange nailing (EN) with a larger size [14], nail dynamization [15], PA [32], and Ilizarov external fixation [16].

### 4.1. Exchange Nail

Concerning mechanical factors, several studies have suggested that NU is caused by a lack of stability. As shown by the studies included in this review, rotational instability (RI) is a cause of NU; Said et al. [11] and later Vaishya et al. [2] focused their attention on the RI that the nail confers to the fixation. Factors that can lead to RI and mechanical failure were broken implants, undersized nail, extensive comminution, incorrect surgical technique, and fracture localization [40]. Vaishya et al. stated that the NU was caused by RI on all the 16 patients examined in his case series [2]. 

To date, EN is universally considered the standard for treating NU of the femoral shaft. It consists of removing the original nail, reaming the femoral canal to stimulate the natural healing response and to allow a larger diameter IMN (at least 1 mm) to be inserted, thereby improving mechanical stability. Despite this, EN can often be difficult to perform due to a lack of specific instrumentation, broken intraosseous screws, broken nails, and damage to the abductor muscles. Furthermore [41], reaming the medullary canal may or may not be advantageous because it can potentially damage endosteal blood vessels, further affecting the biological healing response. Additionally, exchanging the nail with a larger size nail is not applicable if the nail already used is the largest diameter produced by the manufacturer [42].

To confirm this, persistent NU rates after EN ranging from 11.1% to 46% have been reported in the literature [21,43,44]. In a recent systematic review, Vaughn et al. showed a wide range of union rates for this technique (from 28.6% to 100%) [45].

### 4.2. Nail Dynamization

Another low-cost and easy-to-manage therapeutic alternative is nail dynamization. In contrast to these advantages, the disadvantages are the significant instability at the NU site and susceptibility to the shortening of the affected limb. The main risk of dynamization is the possible loss of reduction in subsequent leg length or rotation discrepancies [34], particularly in patients with highly comminuted fractures. In the literature, nail dynamization shows a union rate of approximately 66.4% (24% to 99%) [45].

### 4.3. Ilizarov External Fixation

Another surgical option, although less widely used, for the treatment of femoral shaft NUs is Ilizarov external fixation. Menon et al. reported a high bone union rate in all of the cases treated with the application of the fixator over a long period (about 6 months) [16]. A recent systematic review shows a bone union rate of 60% to 100% in the treatment of long bone NUs of the lower limbs [46]. It is currently seldom used due to poor patient compliance, long treatment and rehabilitation times, and known complications such as pin tract infections and malrotation [47].

### 4.4. Plate Augmentation

In the last two decades, the first cases of patients with femoral shaft NU treated with PA (with or without EN) have begun to appear in the literature; PA improves the biomechanical conditions at the fracture site, removing local rotation instability. 

Analyzing a heterogeneous population of patients and types of NU treated by different authors is not easy. However, the results showed a high bone union rate (98%) among the highest of the various techniques used, in an average time (5.8 months) that is relatively short for a condition that often requires a very long time for full healing.

Confirming the biomechanical aspect, in most of the articles reviewed, the preferred plate-and-screw system was a 4.5-mm compression plate (DCP or DCP), which was rigid enough to resist the rotational forces present in a femoral shaft NU and, when applied in compression mode, could also help limit excessive axial displacement.

Sancheti et al. conducted the largest retrospective study in the literature to date concerning PA in femoral shaft NU, treating them with exchange nailing [31], plate augmentation, and iliac crest bone graft. In this series, 70 out of 70 patients experienced a union after surgery [48]. The analysis of the included articles shows that the rate of bone union in patients undergoing the double procedure, EN + PA in the same operation, is even higher (99.4%) in the face of the increased surgical invasiveness.

Although many studies have shown that PA produces better results and fewer complications than exchange nailing, this technique turns out to be unpopular due to its two main disadvantages [42,43]. Firstly, the surgery can be very invasive: there is a large incision and extensive approach, with significant soft tissue and vascular compromise. Secondly, it requires the compliance of the patient who will experience a limited weight-bearing capacity after the surgery. Another important advantage of PA is the possibility of the debridement of the fracture focus and bone graft; in fact, in as many as 87.4% of cases, the authors of the papers included in the review used a bone graft (without differences between atrophic and hypertrophic NUs).

The limitations of our review are the heterogeneity of the studies included in the review, the retrospective nature of most of these, the lack of control groups, the low volume of data, and the very small sample size in some of the studies. This allows only a descriptive statistical analysis without the possibility of drawing definitive conclusions. 

## 5. Conclusions

The results in a large group of patients show that PA in the treatment of femoral shaft NUs after IMN is a successful option, with a high bone union rate, good functional outcome, and a low complication rate.

Based on these results, PA should find greater space in indications for the treatment of femoral shaft NUs, as it is a technique that offers the surgeon the possibility of increasing mechanical stability and, thanks to the possibility of the bone graft, providing biological support for the NU site.

To standardize the type of treatment in these patients with NU after FSF, randomized controlled clinical trials with homogeneous patient groups and treatments will be needed.

## Figures and Tables

**Table 1:** Studies included in the review and main features.

Author and Year	Type of Study	Cases	Sex (M-F)	Age (Mean)	Localization (P-Mi-D)	Type of NU (A-H)	Bone Graft	Union Rate (%)	Time of Union (m)	Type of Plate	EN	Follow-Up (m)
Choi and Kim, 2005 [20]	Re	15	12-3	36.8	0-10-5	14-1	15	100	7.2	DCP	NS	42.3
Nadkarni et al., 2008 [21]	Re	7	2-5	48.7	3-0-4	NS	7	100	6.8	LCP	NS	14.7
Birjandinejad et al., 2009 [22]	Re	25	NS	31.4	NS	NS	25	100	4.8	DCP	NS	12
Gao et al., 2011 [23]	Re	13	12-1	38.9	NS	NS	13	100	7.5	11 LCP, 2 PHLP	NS	14.4
Hakeos et al., 2011 [25]	Re	7	6-1	42.5	NS	4-3	7	100	5	LC–DCP	NS	17.9
Said et al., 2011 [11]	Re	14	14-0	42	NS	NS	9	100	4.3	DCP	NS	26
Ye and Zheng, 2012 [24]	Re	4	3-1	48.5	2-1-1	2-2	4	100	5	LCP	NS	14.5
Lin et al., 2012 [26]	Re	22	13-9	34.3	5-12-5	13-22	22	100	5.5	DCP	NS	17.2
Khanfour and Zakzouk, 2012 [27]	Re	11	10-1	40	0-0-11	NS	11	100	7.5	DCP	NS	24.2
Park and Yang, 2013 [28]	Re	39	34-5	41.9	8-16-15	30-9	39	97.4	6.1	DCP or LC–DCP	NS	24.8
Wang et al., 2014 [29]	Re	21	9-12	40	NS	NS	21	100	6	LCP	21	12
Jiang et al., 2014 [30]	Re	12	9-3	42	NS	0-12	None	100	4.17	LC–DCP	NS	18.37
Jhunjhunwala et al., 2016 [18]	Re	40	31-9	35	3-32-5	26-14	26	97.5	4	DCP	9	12
Chiang et al., 2016 [17]	Re	30	18-12	50.5	6-13-11	25-5	17 *	96.6	4.3	LC–DCP	NS	NS
Vaishya et al., 2016 [2]	Re	16	11-5	36	NS	4-12	4	100	6.25	LC–DCP	NS	9.6
Sancheti et al., 2017 [31]	Re	70	60-10	40.7	9-48-13	24-46	70	100	4	DCP	70	31.37
Park et al., 2017 [32]	Re	5	5-0	NS	NS	NS	None	100	5.3	LCP	NS	29.5
Verma et al., 2017 [33]	Pr	15	9-6	43.7	NS	NS	15	100	5.2	LCP	15	12.6
Lai et al., 2019 [8]	Re	26	18-8	31.77	NS	26-0	26	88.46	7.5	DCP	NS	11.9
El Zahlawy et al., 2019 [34]	Pr	34	25-9	36.6	7-17-10	NS	34	100	6.3	DCP	NS	12
Uliana et al., 2021 [12]	Re	22	18-4	32.3	1-12-9	12-10	18	86.3	11.7	10 DCP, 12 LCP	NS	23.5
Mittal et al., 2021 [35]	Pr	21	15-6	40	NS	NS	21	100	6	LCP	NS	14.1
Ebrahimpour et al., 2021 [36]	Re	13	NS	42.8	NS	NS	13	92.3	4.75	LCP	NS	12
Mohamed et al., 2022 [37]	Re	20	13-7	32.4	3-7-10	20-0	20	100	4.9	DCP	NS	13

M: male; F: female; P: proximal; Mi: middle; D: distal; NU: nonunion; A: atrophic; H: hypertrophic; m: months; EN: exchange nail; Re: retrospective; Pr: prospective; NS: not specified; DCP: dynamic compression plate; LCP: locking compression plate; LC–DCP: limited contact–dynamic compression plate; PHLP: proximal humeral anatomic locking plate. * With BMPs (bone morphogenic proteins).

**Table 2:** Patient demographics.

Numbers of Patients		502
Age (average on 512 patients)		39.5 ± 5.4 years old
Sex	Male	347 (74.8%)
	Female	117 (25.2%)
	NS	38/502 (7.6%)
NU localization	Proximal	47 (15.0%)
	Middle	168 (53.5%)
	Distal	99 (31.5%)
	NS	188/502 (37.4%)
NU type	Atrophic	200 (61.9%)
	Hypertrophic	123 (38.1%)
	NS	179/502 (35.6%)
First IMN	Anterograde	151 (85.8%)
	Retrograde	25 (14.2%)
	NS	326/502 (65.0%)
Type of plate	DCP	287 (57.1%)
	LCP	109 (21.7%)
	LC–DCP	65 (13.0%)
	PHLP	2 (0.4%)
	DCP or LC–DCP	39 (7.8%)

NS: not specified; NU: nonunion; IMN: intramedullary nailing; DCP: dynamic compression plate; LCP: locking compression plate; LC–DCP: limited contact–dynamic compression plate; PHLP: proximal humeral anatomic locking plate.

**Table 3:** Outcomes.

Numbers of Patients		502
EN before PA		115 (22.9%)
Bone graft	Autologous iliac crest bone graft	437 (87.1%)
Bone union		492 (98.0%)
Time of union (m)		5.8 ± 2.12
Complications	Shortening of the limb	25 (5.0%)
	Wound infections	10 (2.0%)
	Graft site donor pain	3 (0.6%)
	Broken screws	2 (0.4%)
	DVT	2 (0.4%)
	Deep infection	2 (0.4%)
	Infected hematoma	1 (0.2%)
	Total	45 (9.0%)
Follow-up (m)		18.2 (range 9.4–42.3)

EN: exchange nailing; PA: plate augmentation; m: months; DVT: deep venous thrombosis.

## Data Availability

The study data will be available upon request to the corresponding author (email: greco.tommaso@outlook.it).

## Supplementary Materials

The following supporting information can be downloaded at: https://www.mdpi.com/article/10.3390/bioengineering9100560/s1, PRISMA 2020 Checklist.
